# Monocyte-to-high-density lipoprotein cholesterol ratio is associated with the presence and size of thyroid nodule irrespective of the gender

**DOI:** 10.1186/s12944-020-1196-z

**Published:** 2020-03-12

**Authors:** Xing Zhen Liu, Jun Min Wang, Yi Xin Ji, Dong Bao Zhao

**Affiliations:** 1 Hangzhou Aeronautical Sanatorium of Chinese Air Force, Hangzhou, No. 27, Yang Gong Di, Xihu District, Zhejiang, 310007 Hangzhou China; 2 Department of Rheumatology, Changhai Hospital, Naval Military Medical University (The Second Military Medical University), No. 168 Changhai Road, Yangpu District, Shanghai, 200433 China

**Keywords:** Thyroid nodule, Inflammatory markers, Biomarkers

## Abstract

**Background:**

Systemic inflammation may be involved in the formation and progression of thyroid nodule (TN). The aim of this large-scale study was to investigate the association of several simple inflammatory markers with the presence and size of TN.

**Methods:**

A total of 133,698 adults were included for the current analysis. The neutrophil-to-lymphocyte ratio (NLR), platelet-to-lymphocyte ratio (PLR), lymphocyte-to-monocyte ratio (LMR), and monocyte-to-high-density lipoprotein cholesterol ratio (MHR) were calculated. The logistic regression was used to explore the association of the four markers with the presence and size of TN.

**Results:**

The prevalence of TN was 55.1% among females and 44% among males; 13% of women and 8% of men had non-micronodule. In women, MHR and PLR were significantly associated with the presence of TN and non-micronodule; in men, MHR and NLR were significantly associated with the presence of TN and non-micronodule.

**Conclusions:**

As a low-cost, simple, and reproducible inflammatory marker, MHR is strongly associated with the presence and size of TN irrespective of the gender.

## Introduction

Thyroid nodule (TN) are extremely common disorder of the endocrine system, which present in more than 50% of the randomly selected population [1]. Although most TN are benign, about 7–15% are malignant and 5% causing symptoms or thyroid dysfunction [2]. Some well-known factors involved in the formation and progression of TN, such as age, gender, iodine deficiency, and thyroid-stimulating hormone (TSH) [3]. More recently, the role of systemic inflammatory response caused by unhealthy lifestyle such as high calorie intake, sedentary behaviour, and chronic stress in the pathophysiology of TN has also attracted the interest of researchers [4–6].

In clinical practice, C-reactive protein (CRP) and erythrocyte sedimentation rate (ESR) are well established markers for detection the inflammatory conditions [7]. Nevertheless, these traditional inflammatory markers are not widely used in primary care due to their cost, difficulty in interpretating abnormal results, and possible cascade of further tests [8]. Therefore, several simple and cost-effective inflammatory markers, such as neutrophil-to-lymphocyte ratio (NLR), platelet-to-lymphocyte ratio(PLR), lymphocyte-to-monocyte ratio (LMR), and monocyte-to-high-density lipoprotein cholesterol ratio (MHR), have been developed and established links with some tumors and cardiovascular disease [9–11].

Currently, there are no data on the relationship between aforementioned inflammatory indicators and TN. In order to address these research gaps, this large-scale cross section study was conducted to explore the possible association of the four simple and cost-effective inflammatory markers with the presence and size of TN.

## Methods

### Study population

This cross-sectional study was based on the database of subjects who received routine physical examination between November 2015 and January 2019 in China’s Yangtze River Delta region, where is not obvious iodine deficiency area. Subjects with history of other thyroid disease and connective tissue diseases, and those taking amiodarone, glucocorticoid, and estrogens were excluded. Then a total of 133,698 adults with complete data were included in the final analysis. The ethics committee of Hangzhou Aeronautical Sanatorium of Chinese Air Force approved the study protocol.

### Data collection

Basic personal and family medical histories and medication use were collected. Anthropometric indicators [height, weight, waist circumference (WC), and hip circumference (HC)] were measured by well-trained examiners. Systolic blood pressures (SBP) and diastolic blood pressures (DBP) were obtained 3 times on the right arm by trained technicians using a standardized automatic electronic sphygmomanometer. The blood specimens of subjects were collected after a minimum of 8 h of overnight fasting. The peripheral blood cells (e.g., white blood cell, lymphocytes, neutrophils, monocytes, eosinophils, basophils, and platelets) were analyzed using automated hematology system (Beckman Coulter, Inc., USA). (reviewer #1, comment #1) The fasting plasma glucose (FPG) and serum lipid profle [total cholesterol (TC), triglycerides (TG), high-density lipoprotein cholesterol (HDLc), and low-density lipoprotein cholesterol (LDLc)] were assayed using an Automatic Biochemistry Analyzer (HITACHI, Inc., Japan). (reviewer #1, comment #2).

All thyroid ultrasonographic examinations were performed by qualified radiologists using a 5–12 MHz linear probe according to a standard procedure, and carried out on the individuals lying on examination bed with the neck extended. A TN was defned as discrete lesions within the thyroid gland distinct from the surrounding thyroid parenchyma, and which had a solid portion regardless of the presence of a cystic portion. A maximum diameter of ≥3 mm was used to identify the presence of TN and the maximum diameter for TN > 10 mm was defined as thyroid non-micronodule.

### Definitions

BMI was calculated as weight divided by the square of height, and WC divided by the HP was waist to hip ratio (WHR). The calculation of NLR, PLR, and LMR were the division of the corresponding absolute count of blood cells, and MHR was calculated as the ratio of the absolute monocyte count divided by the HDLc.

### Statistical analysis

Statistical analysis was performed using SPSS 18.0 (SPSS Inc.). Data are expressed as numbers (percentage) or means±SD. Categorical variables were analyzed using the chi-squared test, and independent sample t-test were used to compare the differences of continuous variables. Logistic regression analyses was applied to explore the associations of the four inflammatory markers with the presence and size of TN. NLR, PLR, LMR, and MHR were divided into four quartiles, and the lowest quartile was used as a reference (the highest quartile of LMR was used as a reference). The age, genders, obesity, hypertension, and diabetes were adjusted. *P*-value< 0.05 was considered statistically signifcant.

## Results

The general characteristics of individuals are summarized in Table 1. The total of 133,698 subjects included 37.9% women and 62.1% men. The mean age was 46.5 ± 12.9 years and the prevalence of TN was 48.2%. The TN group had higher age, anthropometric indicators, BP, FPG, TC, TG, LDLc, HDLc, and neutrophil count, but lower white blood cell, platelets, lymphocyte, and monocyte than that of non-TN group. (reviewer #1, comment #3).

**Table 1:** General characteristics in the thyroid nodule group and the non-thyroid nodule group

Parameters	Non-TN	TN	*P* value
No., n(%)	69,232(51.8)	64,466(48.2)	< 0.001
Age, y	42.8 ± 11.4	50.4 ± 13.1	< 0.001
BMI (kg/m^2^)	23.6 ± 3.3	23.9 ± 3.2	< 0.001
WC(cm)	79.8 ± 10.0	80.6 ± 9.9	< 0.001
WHR	0.84 ± 0.07	0.85 ± 0.07	< 0.001
SBP (mmHg)	122.3 ± 16.5	125.7 ± 18.1	< 0.001
DBP(mm Hg)	75.7 ± 11.5	76.7 ± 11.7	< 0.001
FPG (mmol/L)	5.65 ± 1.09	5.88 ± 1.33	< 0.001
TC (mmol/L)	4.76 ± 0.89	4.85 ± 0.92	< 0.001
TG (mmol/L)	1.54 ± 1.30	1.57 ± 1.27	< 0.001
LDLc(mmol/L)	2.56 ± 0.74	2.61 ± 0.75	< 0.001
HDLc(mmol/L)	1.48 ± 0.34	1.49 ± 0.35	< 0.001
WBC(×10^9^/L)	6.39 ± 1.55	6.36 ± 1.60	< 0.001
Platelet(×10^9^/L)	204.9 ± 49.8	203.9 ± 51.1	< 0.001
Neutrophil(×10^9^/L)	3.68 ± 1.19	3.69 ± 1.22	0.250
Lymphocyte(×10^9^/L)	2.12 ± 0.58	2.09 ± 0.60	< 0.001
Monocyte(×10^9^/L)	0.39 ± 0.13	0.38 ± 0.12	< 0.001

*TN* Thyroid nodules, *BMI* Body mass index, *WC* Waist circumference, *WHR* Waist to hip ratio, *SBP* Systolic blood pressure, *DBP* Diastolic blood pressure, *FPG* Fasting plasma glucose, *TC* Total cholesterol, *TG* Triglyceride, *HDLc* High-density lipoprotein cholesterol, *LDLc* Low-density lipoprotein cholesterol, *WBC* White blood cell

**Fig. 1:**
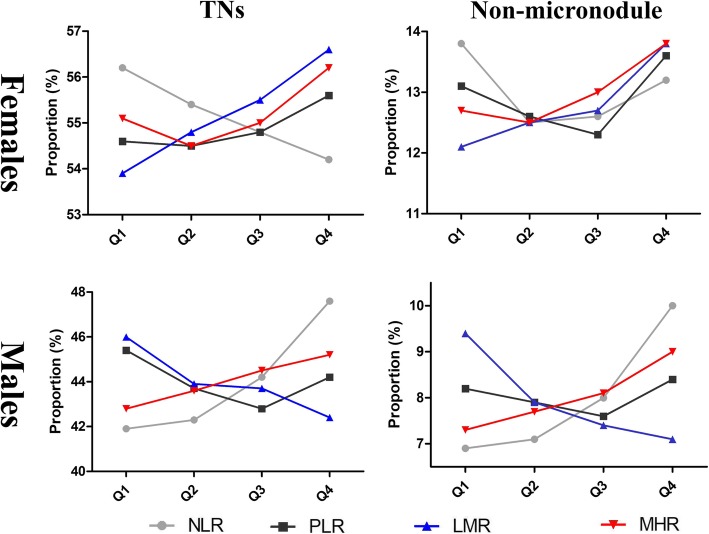
The proportion of TN and non-micronodule by quartiles of the four inflammatory markers in different genders. TN, thyroid nodule; non-micronodule was defined as the maximum diameter for TNs > 10 mm; NLR, neutrophil-to-lymphocyte ratio; PLR, platelet-to-lymphocyte ratio; LMR, lymphocyte-to-monocyte; MHR, monocyte-to-HDL cholesterol ratio

After grouping by gender, the prevalence of TN was 55.1% among females and 44% among males; 13% of women and 8% of men had non-micronodule. The mean values of the four inflammatory markers according to the presence and size of thyroid nodule are presented in Table 2. Among women, the TN group had higher LMR and lower NLR than that of non-TN group, no significant difference in PLR and MHR between the two groups; the non-micronodule group had higher MHR than that of micronodule group, no significant differences in other inflammation indicators between the two groups. Among men, the TN and non-micronodule group all had higher NLR and MHR, but lower LMR than that of non-TN and micronodule group. (reviewer #1, comment #4) Among women, the proportion of TN and non-micronodule showed a significant increase trend as ascending quartiles of PLR, LMR, and MHR (except NLR). Among men, the proportion of TN and non-micronodule showed a significant increase trend as ascending quartiles of NLR and MHR (except PLR and LMR) (Fig. 1).

**Table 2:** The values of the four inflammatory markers according to the presence and size of thyroid nodule

Variable	The presence of TN			The size of TN		
	Non-TN	TN	*P* value	Micronodule	Non-micronodule	*P* value
Females						
NLR	1.89 ± 0.79	1.87 ± 0.79	0.009	1.87 ± 0.79	1.87 ± 0.78	0.920
PLR	112.0 ± 36.9	112.3 ± 37.8	0.434	112.1 ± 37.3	112.6 ± 39.3	0.369
LMR	6.18 ± 2.16	6.27 ± 2.18	< 0.001	6.26 ± 2.15	6.31 ± 2.27	0.099
MHR	5.60 ± 2.25	5.61 ± 2.29	0.688	5.58 ± 2.26	5.72 ± 2.41	< 0.001
Males						
NLR	1.81 ± 0.77	1.88 ± 0.80	< 0.001	1.86 ± 0.77	1.97 ± 0.91	< 0.001
PLR	97.3 ± 31.4	97.4 ± 33.2	0.825	97.2 ± 32.8	98.3 ± 34.9	0.015
LMR	5.73 ± 1.96	5.63 ± 1.98	< 0.001	5.66 ± 1.98	5.49 ± 1.99	< 0.001
MHR	8.00 ± 3.22	8.11 ± 3.24	< 0.001	8.06 ± 3.22	8.33 ± 3.33	< 0.001

TN, thyroid nodules; non-micronodule was defined as the maximum diameter for TNs > 10 mm; *NLR* Neutrophil-to-lymphocyte ratio, *PLR* Platelet-to-lymphocyte ratio, *LMR* Lymphocyte-to-monocyte, *MHR* Monocyte-to-HDL cholesterol ratio

**Fig. 2:**
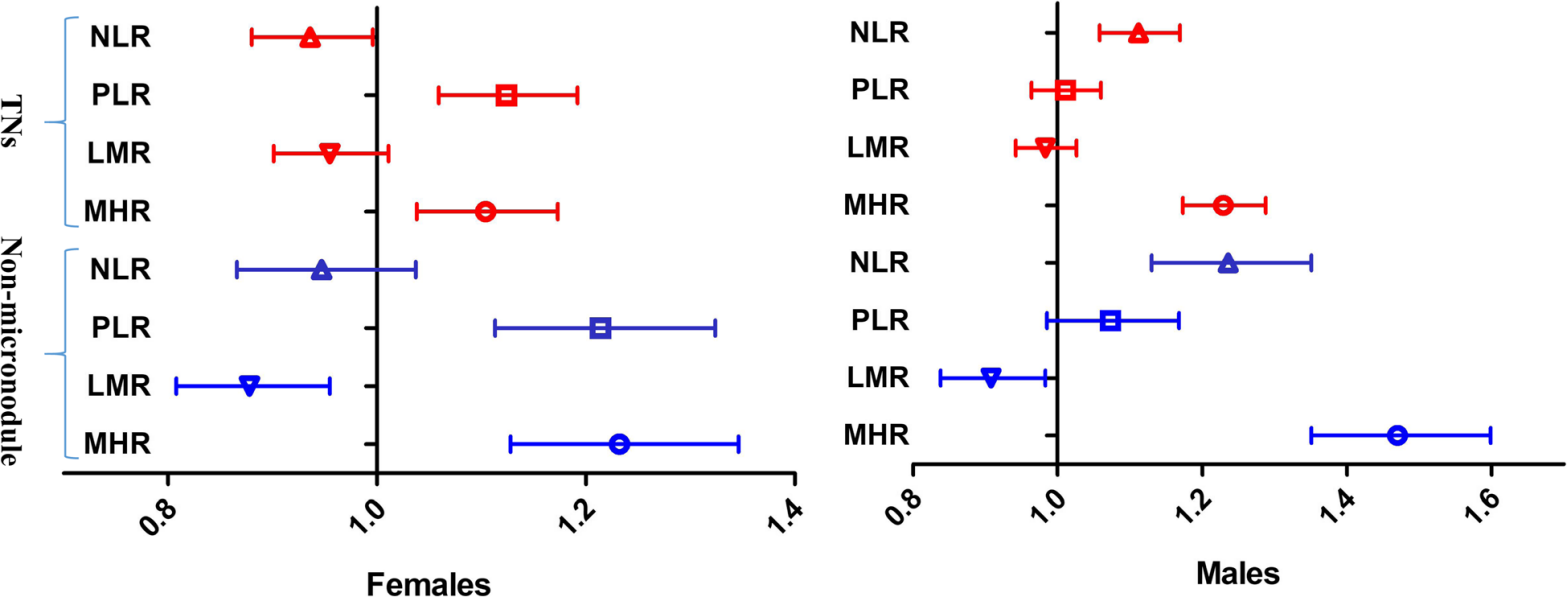
Multivariate-adjusted odds ratios (ORs) of TN and non-micronodule with the highest quartile of the four inflammatory markers in different genders. TN, thyroid nodule; non-micronodule was defined as the maximum diameter for TNs > 10 mm; NLR, neutrophil-to-lymphocyte ratio; PLR, platelet-to-lymphocyte ratio; LMR, lymphocyte-to-monocyte; MHR, monocyte-to-HDL cholesterol ratio

Multivariate-adjusted odds ratios (ORs) of TN and non-micronodule with the highest quartile of the four inflammatory markers are shown in Fig. 2. MHR and PLR in women, and MHR and NLR in men had significant ORs for TN and non-micronodule. In women, the ORs in the fourth quartile of PLR for TN and non-micronodule were 1.124(95%C.I. 1.059–1.192) and 1.214(95%C.I. 1.113–1.324); the ORs of MHR for TN and non-micronodule were 1.104(95%C.I. 1.038–1.173) and 1.232(95%C.I. 1.128–1.346); and all *P* < 0.001. In men, the ORs in the fourth quartile of NLR for TN and non-micronodule were 1.112(95%C.I. 1.058–1.169) and 1.236(95%C.I. 1.130–1.351); the ORs of MHR for TN and non-micronodule were 1.229(95%C.I. 1.173–1.288) and 1.470(95%C.I. 1.351–1.599); and all *P* < 0.001.

## Discussion

This large-scale cross-sectional study was designed to determine the relationship between four simple inflammatory indicators and the presence and size of TN. The results of this investigation show that MHR were significantly associated with the presence and non-micronodule of TN in both genders, but PLR and NLR were significantly associated with the presence and non-micronodule of TN in women and men, respectively.

The TN area frequently observed condition in routine physical examination and more frequent in females than in males. In this study, the prevalence of TN was 55.1% among females and 44% among males, which is consistent with those of many previous studies [12, 13]. TN are closely related to components of the metabolic syndrome such as abdominal obesity, hypertension, hyperglycemia, and dyslipidemia [14, 15]. Therefore, not surprisingly, the TN group also had higher mean values of anthropometric indicators, BP, FPG, TC, TG, and LDLc than that of non-TN group in the present study. (reviewer #1, comment #4) Considering the close association of obesity and metabolic abnormalities with inflammation markers [16, 17], we adjusted BMI, WC, hypertension, dyslipidemia, and diabetes during regression analysis to reduce the impact of these basic disease states on the relationship between those inflammation markers and TN.

One unanticipated finding was that the TN group had higher HDLc level and lower values of peripheral blood cells than that of non-TN group. However, when grouped by gender, some inflammatory indicators composed of these indicators in the TN and non-micronodule group were significantly higher than the corresponding control group. For example, except for MHR in the TN group in women, MHR were significantly higher in the TN and non-micronodule group. These results show that the combination of some simple clinical indicators can better reflect their correlation with some diseases. In addition, gender disparity may be a key factor that must be considered when exploring the correlation between these inflammatory indicators and thyroid disease. (reviewer #1, comment #4).

The traditional inflammatory markers (CRP and ESR) are recommended for the assessment of rheumatic diseases, infectious diseases, and several cancers in clinical practice [18, 19]. But the cost, difficulty in interpretating abnormal results, and possible cascade of further tests of the traditional inflammatory markers limit their clinical application. Conversely, the novel inflammatory markers (NLR, PLR, LMR, and MHR) are simply calculated from the leukocyte subsets, platelet counts, or HDLc which are routinely checked, inexpensive, readily available biomarkers. So these novel inflammatory markers have a wider range of applications, especially in primary health institutions.

In the leukocyte subsets, neutrophilia reflect a low-grade inflammation stage and lymphopenia represent a physiological stress and poor general health [20, 21]. So NLR reflects both inflammation and stress response which are two important immune pathways. NLR has been investigated in several studies on cancer [22], cardiovascular disease [23], and thyroid disease [24, 25]. But only one study so far had investigated the relationship between NLR and TN, which showed a negative association between high values of NLR and the presence or size of the nodules [26]. In our study, we found a positive association of NLR with the presence and size of TN in men but not in women. This gender disparity may be related to the effects of sex hormones on lymphocytes [27].

Apart from the role in blood coagulation and hemostasis, platelets also play a key role in modulating inflammatory reactions, including lymphocyte functioning. The cross-talk between blood platelets and lymphocyte have a potent effect on thyroid gland diseases [28]. So several attempts have been made to explore the connections between PLR and thyroid tumors and thyroiditis [29, 30]. However, until now, no reported research has focused on the association between PLR and TN. Our study demonstrated that an elevated values of PLR was associated with the the presence and size of TN only in women but not in men. The effects of sex hormones on platelet and lymphocyte function may partially explain the gender differences of the relationship of PLR with TN [27, 31].

In the pathophysiology of tumors, lymphocytes play an important role in anti-tumor effects and lymphopenia produces an insufficient immune response against a tumor. So lower LMR was originally used to assess the prognosis of some tumors [32]. In the near future, lower LMR was also demonstrated to be associated with metabolic syndrome and vascular disease [33]. Until now, there is no research about the relationship between LMR and TN. In this study, we observed that lower LMR value was not significantly associated with TN in both men and women. The reason for the negative result is not clear but it may have something to do with the multiple roles of lymphocytes. In addition to anti-tumor effects, lymphocytes are also involved in the production of thyroid autoantibodies, which not only causes destruction but also stimulates proliferation of thyroid cells through TSH receptor activation [27, 34].

Monocytes are critical defense components a bridge between the innate and adaptive immune responses. As a source of various cytokines and molecules, monocytes are also important participants in the inflammatory pathway [35]. Contrarily, HDLc can counteract the pro-inflammatory and pro-oxidant effects of monocytes [36]. So the combination of monocytes and HDLc as a single ratio is promising to be a qualified indicator of oxidative stress and inflammation. Different from the above three indicators, most of the existing research focuses on the relationship between MHR and atherosclerosis [37]. So this study is also the first to explore the association between MHR and TN, and we found MHR was significantly associated with the presence and non-micronodule of TN in both genders. The good performance of MHR raise intriguing questions regarding the close relationship between inflammation, metabolic disorders, and TN.

To the best of our knowledge, this might reportedly be the first large-scale study demonstrating the association between the simple inflammatory markers and the presence and size of TN. But some limitations of our study should also be mentioned. First, because of the retrospective cross-sectional design, we can not show a causal association between the four inflammatory markers and TN. Second, the study population might limit the generalisability of the results to other ethnic groups. Third, less similar research resulting in a limited possible comparisons.

## Conclusions

MHR is strongly associated with the presence and size of TN irrespective of the gender. But there is a gender difference in the association of NLR and PLR with TN. These findings not only contribute in several ways to our understanding of the relationship between TN and inflammation, but also provide some candidates of simple, practical, and cost-effective inflammatory markers in the prevention and management of TN. However, further prospective and randomized studies are needed to confirme our findings.

## Data Availability

The datasets used and/or analyzed during the current study are available from the corresponding author on reasonable request.

## Abbreviations

CRP: C-reactive protein
ESR: Erythrocyte sedimentation rate
LMR: Lymphocyte-to-monocyte ratio
MHR: Monocyte-to-high-density lipoprotein cholesterol ratio
NLR: Neutrophil-to-lymphocyte ratio
PLR: Platelet-to-lymphocyte ratio
